# Outcome of critically ill patients with haematological malignancy admitted to the ICU as an emergency

**DOI:** 10.1186/cc12454

**Published:** 2013-03-19

**Authors:** T Lofaro, S Easdale, S Rowe, M Ostermann, R Carr

**Affiliations:** 1 Queen Elisabeth Hospital, Woolwich, UK; 2 Guys' and St Thomas' Hospital, London, UK

## Introduction

ICU admission policies regarding patients with haemato-logical malignancy still vary despite data showing an acceptable prognosis. Our aim was to review ICU and 6-month outcomes in this group when requiring emergency admission to the ICU in a tertiary cancer centre.

## Methods

A retrospective review of medical notes between 2004 and 2012.

## Results

A total of 249 patients were admitted, of whom 54 had more than one admission. There were 310 episodes in total. Leukaemia *n *= 85; lymphoma *n *= 90; myeloma *n *= 36. We compared the characteristics of those who survived ICU admission with those who failed to survive to discharge from ICU. The two populations were similar (age 51 vs. 57; males 59% vs. 57%). Those who survived had a lower APACHE II score on admission (19 vs. 23; *P *0.001), lower mean organ failure scores (1 vs. 2; *P *0.05), lower requirements of inotropes (26% vs. 50%; *P *= 0.001), ventilation (31% vs. 64%; *P *= 0.001) and filtration (11% vs. 26%; *P *= 0.004). There was no difference in the prevalence of sepsis at the time of admission (64% vs. 70%). Both groups included patients with prior bone marrow transplant (38% vs. 40%). Of note, ICU and 6-month survival were 27% and 50%, respectively. These values are lower than those reported in the literature to date.

## Conclusion

ICU and 6-month mortalities were 27% and 50%, respectively. Patients with haematological malignancy stand to benefit from intensive care, and should be offered admission based on clinical need.
